# miR-361-5p as a promising qRT-PCR internal control for tumor and normal breast tissues

**DOI:** 10.1371/journal.pone.0253009

**Published:** 2021-06-08

**Authors:** Sogol Ghanbari, Adel Salimi, Saeid Rahmani, Nahid Nafissi, Ali Sharifi-Zarchi, Seyed Javad Mowla

**Affiliations:** 1 Molecular Genetics Department, Biological Sciences Faculty, Tarbiat Modares University, Tehran, Iran; 2 Computer Engineering Department, Sharif University of Technology, Tehran, Iran; 3 Surgical Department, School of Medicine, Iran University of Medical Sciences, Tehran, Iran; Fondazione IRCCS Istituto Nazionale dei Tumori, ITALY

## Abstract

**Background:**

One of the most widely used evaluation methods in miRNA experiments is qRT-PCR. However, selecting suitable internal controls (IC) is crucial for qRT-PCR experiments. Currently, there is no consensus on the ICs for miRNA qRT-PCR experiments in breast cancer. To this end, we tried to identify the most stable (the least expression alteration) and promising miRNAs in normal and tumor breast tissues by employing TCGA miRNA-Seq data and then experimentally validated them on fresh clinical samples.

**Methods:**

A multi-component scoring system was used which takes into account multiple expression stability criteria as well as correlation with clinical characteristics. Furthermore, we extended the scoring system for more than two biological sub-groups. TCGA BRCA samples were analyzed based on two grouping criteria: Tumor & Normal samples and Tumor subtypes. The top 10 most stable miRNAs were further investigated by differential expression and survival analysis. Then, we examined the expression level of the top scored miRNA (hsa-miR-361-5p) along with two commonly used ICs hsa-miR-16-5p and U48 on 34 pairs of Primary breast tumor and their adjacent normal tissues using qRT-PCR.

**Results:**

According to our multi-component scoring system, hsa-miR-361-5p had the highest stability score in both grouping criteria and hsa-miR-16-5p showed significantly lower scores. Based on our qRT-PCR assay, while U48 was the most abundant IC, hsa-miR-361-5p had lower standard deviation and also was the only IC capable of detecting a significant up-regulation of hsa-miR-21-5p in breast tumor tissue.

**Conclusions:**

miRNA-Seq data is a great source to discover stable ICs. Our results demonstrated that hsa-miR-361-5p is a highly stable miRNA in tumor and non-tumor breast tissue and we recommend it as a suitable reference gene for miRNA expression studies in breast cancer. Additionally, although hsa-miR-16-5p is a commonly used IC, it’s not a suitable one for breast cancer studies.

## Introduction

Identifying dysregulated genes involved in the carcinogenesis and tumor progression is an important component in cancer research [1]. Recently, high-throughput sequencing techniques is being used to conduct the whole transcriptome profiling, however, the main molecular diagnosis tests in clinic still relies on the cheaper quantitative real-time RT-PCR technique [2]. qRT-PCR is one of the most reliable and powerful tools which promises high specificity, sensitivity, and reproducibility to precisely detect the changes in gene expressions in a broad range of clinical samples, collected under different conditions [3, 4]. However, the precision of the results largely depends on choosing correct internal control to normalize the data [5]. Ideally, the expression of a reliable internal control (IC) gene should not be altered in tested tissues or cells under experimental conditions. The ideal internal control gene is universally valid, with a stable expression level across all tissue samples, cells, and experimental treatments. Although such an ideal IC has not yet been found [6, 7].

miRNAs represent an important new class of regulatory biomolecules that play fundamental biological roles including development, differentiation, apoptosis, and metabolism. Over the past decade numerous studies have been done on misregulation of miRNAs expression in various cancers, and the suitability of those miRNAs as novel biomarkers to diagnose, classify, prognose and treat patients with cancer [8, 9]. To use miRNAs as a biomarker in the clinic, it is crucial to standardize miRNA testing and make it reliable and reproducible in routine diagnostic applications. Over the past several years, some technical approaches had been employed to quantify miRNAs in clinical samples, among them, qRT-PCR has become the most popular one. Because of its sensitivity and specificity, it can detect low copy number of precursor and mature miRNA [10].

There is currently no consensus on suitable ICs for quantitative analysis of miRNA in human breast tissue. It is also clear that the traditionally used *GAPDH* and *β-actin* (*ABTB*) house-keeping genes are less validated as suitable ICs for miRNAs quantification [11, 12]. Ribosomal RNAs are another choice, however, they are expressed at much higher levels than target RNAs, making it difficult to normalize rare transcripts and rRNAs at same biological or clinical samples [13]. Additionally, although small-nucleolar RNAs such as RNU6, RNU6B and RNU48 are frequently used as reference genes, there existed evidence that snoRNAs can introduce some bias to miRNA expression in cancer studies [14]. Moreover, it has been argued that miRNAs must be normalized with internal genes from the same RNA class [12, 15]. Up to date, only a few candidate reference miRNAs (miR-16 and let-7) have been reported for breast tissue miRNA quantification [11, 12, 14, 15]. Conversely there has been evidence about the role of aforementioned ICs in cell differentiation and carcinogenesis [16–18] reinforcing the need for a better miRNA IC. To choose a study-specific IC, usually, a panel of 5–20 ICs are tested using qRT-PCR and analyzed by tools like Normfinder, geNorm and BestKeeper [5, 19, 20]. However, this approach is limited to a small number of ICs and the logic behind the assumption of these methods is that the mean expression of ICs is constant between samples which is not satisfied when the number of ICs is too low in a qRT-PCR experiment. Additionally, most researchers simply choose their IC based on literature [21].

Thanks to accession to the total read count, whole transcriptomic RNA-Seq data is a great source to discover stably expressed reference genes. Unlike qRT-PCR data, within samples technical variation can be normalized without control genes. Some studies have taken advantage of this source of data to identify stably expressed reference genes in various diseases, and have reported its adequacy for this purpose [22–24].

This study aimed to find the most suitable references for normalizing qRT-PCR data on miRNA expression in breast tissues, using a multi-component scoring system on miRNA-Seq data. Our scoring method takes into account multiple expression stability criteria as well as finding their correlation with clinical aspects of the samples. The top 10 most stable and promising miRNAs were introduced and evaluated. hsa-miR-361-5p was the best miRNA in overall scores for both tumor & normal samples as well as tumor subtype grouping criteria. We validated our findings using qRT-PCR assay and revealed hsa-miR-361-5ps superiority over hsa-miR-16-5p and U48.

## Materials & methods

### miRNA-Seq dataset

The Cancer Genome Atlas (TCGA) is a landmark cancer genomics program which includes molecular datasets for various type of cancer tissues [25]. The breast cancer (BRCA) miRNA expression data of TCGA was obtained via TCGAbiolinks package v2.12.3 [26] specifying data type as Isoform Expression Quantification. 1097 tumor tissue and 104 adjacent normal tissue samples were obtained. miRNA names were annotated by miRBase v21 using miRBaseConverter v1.8 [27]. CPM (read counts per million) values were used as a measure of expression level of miRNAs in stability analysis.

### Multi-component reference gene scoring system

A multi-component scoring system introduced by Krasnov et al. [28] was used in the context of miRNA expression. We modified and extended it in order to give it the ability of handling more than two subgroups. This system consists of several scoring components *S*_*i*_ which examine gene’s expression value and its dispersion in subgroups and pooled samples, as well as correlation with clinical and pathological features of patients. Detailed description of each component is presented in Table 1. Overall expression stability score *S*^*exp*^ is calculated as weighted geometric mean of scoring components Eq (1).

**Table 1:** Components of scoring function (obtained from associated paper).

Component	factor	Variable (x = …)*	IV	IP	CS	Sq	CA	W	Number of Times applied
S_DP_	T-N expression level difference (pooled samples)	Abs (log_2_FC _p_) _10–90_	0.05	0.25	2.5	1	0	4	1 (all samples)
S_DL_	T-N expression level difference (paired samples)	Abs (Average(log_2_FC_L_) _10–90_)							1 (paired samples)
S_DoO_	T-N expression level difference: outliers, overexpression	Abs (Average(log_2_FC_L_) _90–100_)	0.1	0.7	2.5	1	10	1	1 (paired samples)
S_DoU_	T-N expression level difference: outliers, underexpression	Abs (Average(log_2_FC_L_) _0–10_)							1 (paired samples)
S_DLc_	Cumulative T-N expression difference among paired samples	Average (Abs(log_2_FC_L_) _10–90_)	0.1	0.5	2.5	1	5	2	1 (paired samples)
S_EStD_	Expression level stability: standard deviation	StDev (CPM) _10–90_ /Average(CPM) _10–90_	0.1	0.3	2	1	5	1.5	2 (all samples: normal and tumor)
S_EoH_	Expression level stability: outliers (high expression)	log_2_ (Average(CPM) _90–100_ /Average (CPM) _10–90_)	0.1	0.7	2.5	1	5	0.75	2 (all samples: normal and tumor)
S_EoL_	Expression level stability: outliers (low expression)	log_2_ (Average(CPM) _10–90_ /Average (CPM) _0–10_)							2 (all samples: normal and tumor)
S_EA_	Average expression level	1/log_2_ (CPM) _10–90_	0.30**	0.43**	3	1	0	6	1 (all tumor samples)
S_Cp_	Correlations of expression with clinical parameters (p-values)	-log_2_ (p-value)	2	4	3	0.3	5	0.3	15 (3 × 5; 3: CPM_10-90_ all tumor samples, CPM_10-90_ all normal samples, (log_2_FC_L_)_10-90_; 5: pathologic TNM classification, pathologic stage, neoplasm cancer status)
S_Cr_	Correlations of expression with clinical parameters (r_s_)	Abs (r_s_)	0.1	0.25	2.5	0.3	5	0.2	15 (the same as above)

IV, ideal value; IP, inflection point; CS, curve slope; Sq, “squeeze”; CA, constant add; W, weight; Abs (…), absolute value; Average (…), mean value; CPM, counts per million, gene expression level; FCP, ratio of the average CPM in a pool of tumor samples to the average CPM in a pool of normal samples; FCL, ratio of CPM values between tumor and matched normal tissue (per each paired sample); StDev (…), standard deviation; rs, Spearman’s correlation coefficient.

* Percentiles, which were taken into calculation, are indicated as a subscript.

** These two parameters are different from the associated paper. as we filtered out miRNAs based on their expression level threshold of 5.9 CPM, here IV and IP were set as 1/log(10) and 1/log(5).

$$S^{Exp}={\left(\overset{N}{\underset{i=1}{\prod }}{\left(S_{i}+CA_{i}\right)}^{W_{i}}\right)}^{1/\sum_{i=1}^{N}W_{i}}$$ (1)

Here: *W*_*i*_ specifies each component’s importance.

*CA*_*i*_ is a constant to prevent zero values of *S*_*i*_ from making whole expression zero.

*N* is the number of components.

Each component is calculated based on a parameterized (1-sigma)-like function as in Eq (2):

$$S_{i}=\frac{100}{1+S_{q}+{\left(\frac{\text{max}\left(x-IV;0\right)}{IP-IV}\right)}^{CS}}$$ (2)

Detailed description can be found in the associated paper [28].

### Extended scoring system for more than two subgroups

In order to extend the scoring system for multiple subgroups, following manipulations were carried out:

1. Components associated with paired samples were removed.
2. For *S*_*DP*_, logFC was calculated between every two subgroups of the data.
3. Beside *S*_*EA*_, all other scoring components were applied to each subgroup separately and the weights were distributed according to the number of subgroups.

### Sample collection

Primary breast tumor tissues (n = 68) were obtained from patients undergoing surgery, at Khatam-al-Anbia and Rasoul-Akram Hospitals, Tehran, Iran. This research involved collecting human tissues with no experimenting on human subjects or animals. In vitro experiments on commercial cell lines and pathological samples were approved by Ferdowsi University of Mashhad (code number: IR.UM.REC.1399.104). Samples were categorized into 34 pairs of breast tumor and their adjacent apparently normal tissues from October 2018 to June 2019. The studied specimens were examined by pathologists and classified according to the standard histopathological parameters. Tissues were immediately snap-frozen in liquid nitrogen and stored at -80°C until RNA extraction. Clinicopathologic characteristics of patients are summarized in Table 2 as well as S1 Table.

**Table 2:** Clinicopathologic characteristics of 34 breast cancer patients.

Age (mean ± SD)	50 ± 10	TNM classification	Cases (n)
**Tumor Subtype**	**Cases (n)**	** **T = 1	13
luminal A	22	** **T = 2	12
luminal B	2	** **T = 3	3
Her2 Overexpressed	2	** **Undefined T	6
TNBC	3	** **N = 0	17
Undefined Subtype	5	** **N = 1	5
**Grade**	**Cases (n)**	** **N = 2	5
I	4	** **N = 3	1
II	16	** **Undefined N	6
III	9	** **M = 0	34
Undefined Grade	5		
**Stage**	**Cases (n)**		
I	9		
IIA	10		
IIB	3		
IIIA	5		
IIIC	1		
Undefined Stage	6		

T, N and M refer to the primary tumor size, nodal status and distant metastases status according to the TNM classification system. TNBC, Triple Negative Breast Cancer.

### Total RNA isolation

Total RNA was isolated from all samples (approximately 100 mg) using the RiboEx Total RNA reagent (GeneAll Biotechnology, South Korea). The amount of extracted RNA was quantified by measuring the absorbance at 260 nm. The purity of the RNA was determined by calculating the ratio of the absorbance at 260 and 280 nm. The absence of degradation of the RNA was confirmed by electrophoresis of the RNA on a 1% agarose gel containing ethidium bromide.

### Polyadenylation and reverse transcription

For the S-Poly(T) method, extracted total RNA was polyadenylated with Poly(A) Polymerase Tailing Kit (New England Biolabs., UK., Ltd.). Briefly, a 10 μl reaction including 1 μg total RNA, 1 μl of 10 × reaction buffer, 1 μl of 10 mM ATP and 1 unit of Poly(A) polymerase was incubated at 37°C for 30 min, followed by enzyme inactivation at 65°C for 5 min. After polyadenylation, reverse transcription was performed in a 20 μl reaction containing 10 μl of the polyadenylation reaction product, 2 μl of Anchored Oligo(dT), 1 μl of RiboLock RNase Inhibitor (20 U/μL), 4 μl of 5X Reaction Buffer, 2 μl 10 mM dNTP Mix, and 1 μl of RevertAid M-MuLV RT (200 U/μL) (Thermo Fisher Scientific., UK). The reaction was incubated at 42°C for 70 min and then terminated by heating at 70°C for 5 min.

### RT-PCR and real-time qRT-PCR

PCR assays were performed using the primers listed in Table 3. All oligonucleotides were analyzed for potential secondary structure and dimerization using OligoAnalyzer 3.1. qRT-PCR was performed on a StepOne Plus System (Applied Biosystems) using Power EVA Green PCR Master Mix (BIOFACT Co., Ltd., Korea). PCR was performed using the following protocol: initial denaturation 95°C for 5 min, then 40 cycles at 95°C for 15 s, 60°C for 20 s, 72°C for 20 s. To verify that the used primer pair produced only a single product, a DNA melting curve analysis was utilized after thermocycling, determining dissociation of the PCR products from 60 to 95°C (with a heating rate of 0.3°C and continuous fluorescence measurement). All the qRT-PCR reactions were carried out in triplicate for each sample. The p-values obtained for five cDNA dilutions (1:1, 1:2, 1:4, 1:8, 1:16).

**Table 3:** qRT-PCR primers.

Gene	Accession number	Primer	Band size	Sequence (5’-3’)
hsa-miR-16-5p	MIMAT0000069	Forward	72 bp	GCGGGTAGCAGAACGTAAATA
hsa-miR-361-5p	MIMAT0000703	Forward	71 bp	GGCGTTATCAGAATCTCCAGG
hsa-miR-21-5p	MIMAT0000076	Forward	70 bp	CCGGCCTAGCTTATCAGACTG
SNORD 48	NR_002745	Forward	122 bp	TGACCCCAGGTAACTCTGAGTGTGT
		Universal Reverse primer		AACTCAAGGTTCTTCCAGTCACG
		Anchored Oligo dT mix		GCGTCGACTAGTACAACTCAAGGTTCTTCCAGTCACGACGTTTTTTTTTTTTTTTTTT(V)

The comparative threshold cycle (Ct) was determined for each miRNA and the relative amount of each miRNA in individual samples were described as ΔCt (Ct miRNA- Ct internal control). ΔCt values were employed for expression level comparison of miRNAs in control vs. cancerous samples.

### Primer validation

The amplification efficiency of all primer pairs varied from 80% to 99% and the coefficient of determination (R2) ranged between 0.794 and 0.983. Single peaks were observed for the products of all primer pairs according to the melting curve analysis, and the sequences of the amplified DNA fragments matched the sequences of the reference and target genes in GenBank.

### Statistical analysis

All statistical analysis were executed in RStudio integrated development environment v1.2.5033 and R language v3.6.1 [29]. Differential Expression analysis was performed using limma+voom package v3.40.6 [30, 31]. Benjamini-Hochberg adjusted p-value of 0.05 was set as statistical significance threshold. The web tool miRPower [32] which performs survival analysis and provides Kaplan-Meier plots was utilized with dataset as METABRIC [33] with 1262 breast tissue samples to evaluate ICs in terms of their association with prognostic features. Low- and high-risk groups were split based on median expression. Other figures were provided using ggplot2 v3.2.1 [34] and fmsb v0.7.0 [35] packages.

## Results

### Most stable miRNAs in breast tissue based on multi-component scoring system

**Fig 1:**
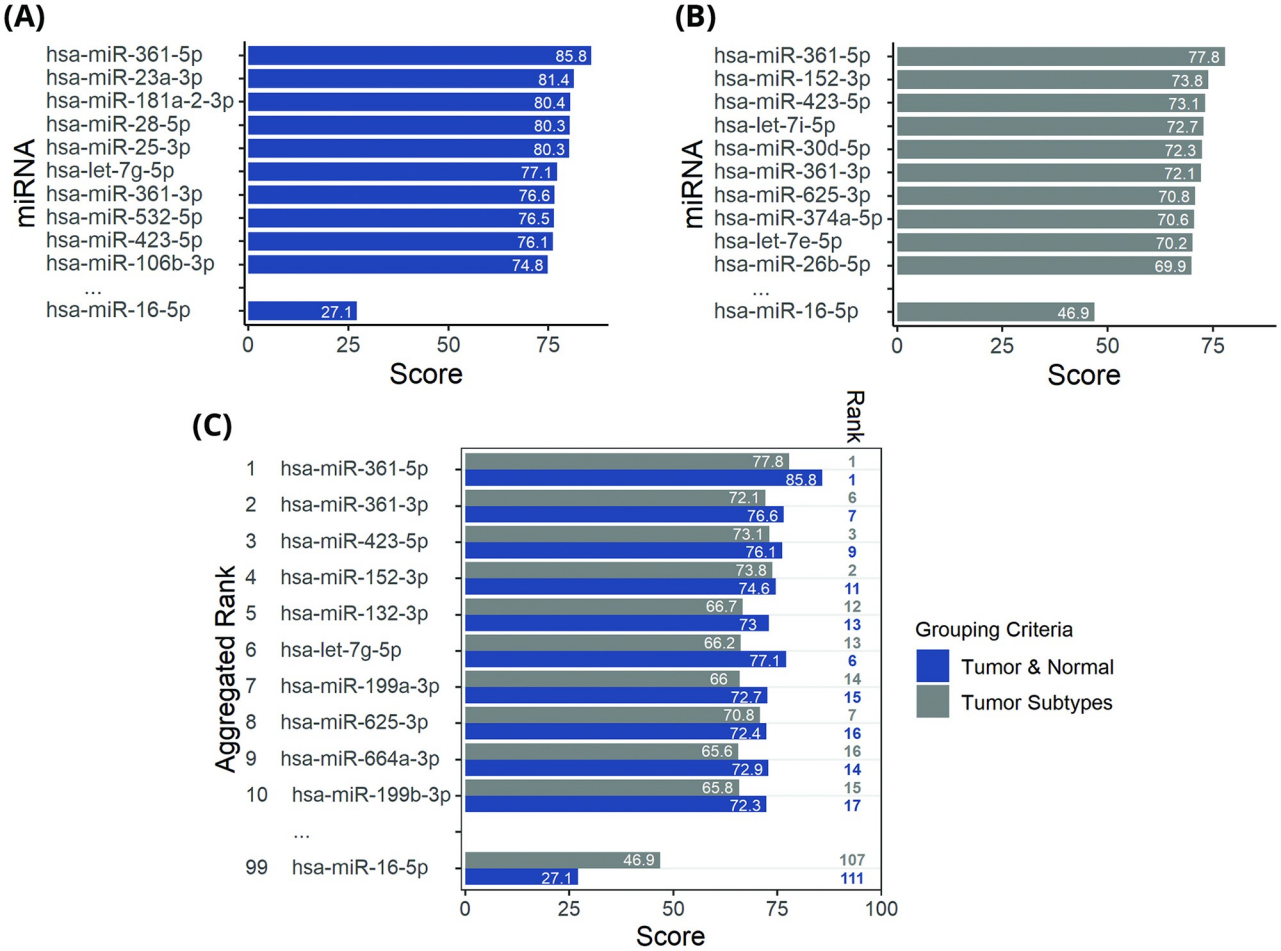
Multi-component scoring results for different grouping criteria. (A) for Tumor & Normal grouping criterion and (B) Tumor Subtypes. (C) is the aggregated Rank results using scorings in (A) and (B). The Rank column in (C) represents miRNAs rank in that grouping criterion. All scores are out of 100.

**Fig 2:**
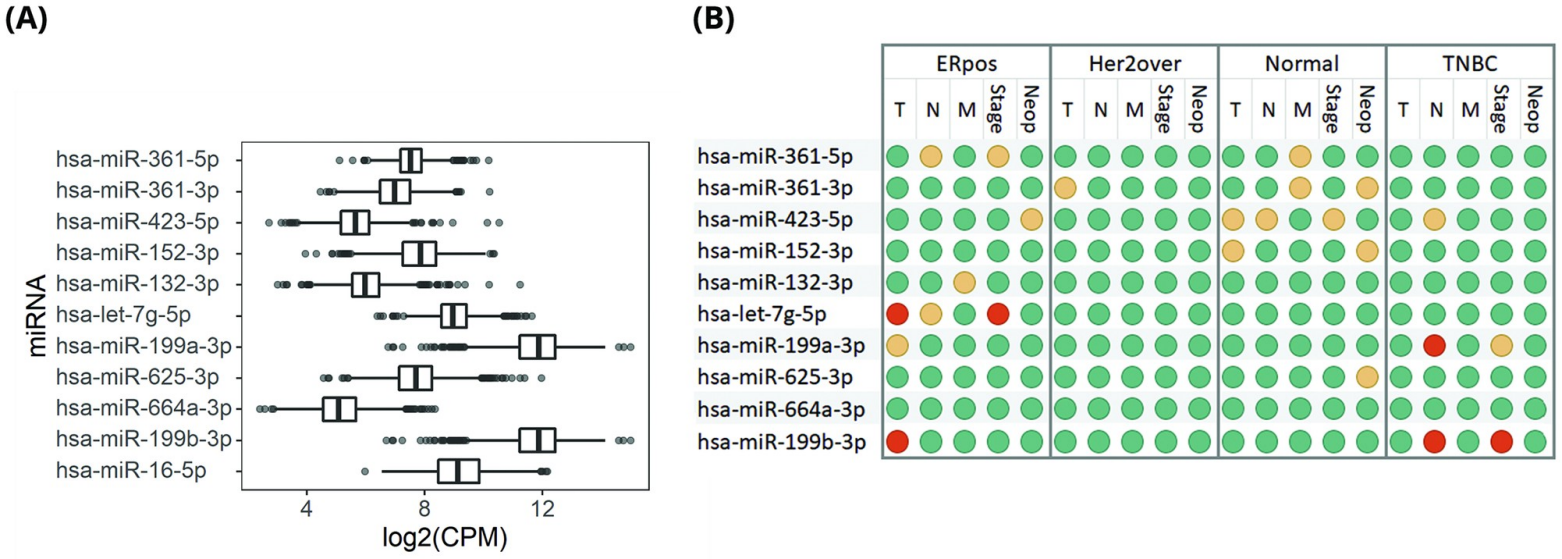
Expression level and correlation with clinical aspects in top 10 most stable miRNAs. (A) Expression level of top 10 most stable miRNAs + hsa-miR-16-5p for comparison. CPM, Count Per Million. (B) most stable miRNAs correlation with clinical aspects in various biological subgroups. Colors represent statistical significance: red, p<0.01; yellow, 0.01<p<0.1; green, p>0.1. ERpos, Estrogen positive; Her2over, Her2 Overexpressed; Normal, adjacent Normal Tissue; TNBC, Triple Negative Breast Cancer; T, N and M are TNM classification indexes; Stage, pathological Stage; Neop, Neoplasm Status.

Starting our work on TCGA miRNA expression data (104 normal and 1097 tumor samples of breast), we first filtered out miRNAs which had expression levels less than 5.9 CPM (count per million, equivalent to average read count of 20) in more than 5 percent of samples. Using this filter, 185 miRNAs remained for further analysis. In order to obtain the most promising and stable internal miRNA controls we ran our multi-component scoring system on two different grouping criteria. Firstly, based on Tumor & Normal samples and secondly based on Tumor Subtypes. Tumor Subtypes were defined as: Estrogen receptor status positive (n = 795), Her2 receptor over expressed (n = 39) and Triple Negative Breast Cancer (n = 125; Fig 1). The hsa-miR-361-5p had the highest stability score in both grouping criteria, reaching scores of 85.7 and 76.1 out of 100. By using Robust Ranking Aggregation (RRA) [36] we aggregated rankings of the two grouping criteria. According to the aggregated ranks, hsa-miR-361-3p, hsa-miR-423-5p and hsa-miR-152-3p were the best ICs after hsa-miR-361-5p. Expression level of top 10 miRNAs along with hsa-miR-16-5p are represented in Fig 2A. Among them, hsa-miR-199a-3p and hsa-miR-199b-3p were the most abundant miRNAs.

### Correlation with clinical and pathological characteristics

In each biological subgroup, Spearman’s correlation coefficient was calculated between the most stable miRNA expression and the following 5 features: TNM (Tumor, Node, and Metastasis) classification indexes, pathologic stage and follow-up person neoplasm cancer status. The most significant correlations were as follows: hsa-let-7g-5p with Tumor (p≤0.001) and pathologic stage (p≤0.001) and hsa-miR-199a-3p with Node (p≤0.005; Fig 2B).

### Differential expression analysis

A differential expression analysis for Tumor vs Normal samples was performed on TCGA BRCA miRNA-Seq data considering both paired samples and all samples. As presented in Table 4, although there are some significant differentiations in the most stable miRNAs, their abs (logFC) are smaller than 0.32.

**Table 4:** Differential expression analysis for Tumor vs Normal samples on TCGA breast cancer miRNA-Seq data.

	logFC	adj.P.Val	logFC_paired	adj.P.Val_paired	Mean Exp.
hsa-miR-361-5p	-0/13	0/05	-0/31	**1/38E-05**	726
hsa-miR-361-3p	-0/15	0/11	-0/11	0/31	535
hsa-miR-423-5p	-0/11	0/20	-0/26	**0/01**	193
hsa-miR-152-3p	-0/31	**0/0005**	-0/21	**0/02**	905
hsa-miR-132-3p	-0/18	**0/03**	-0/32	**0/006**	284
hsa-let-7g-5p	0/11	0/07	0/06	0/5	1849
hsa-miR-199a-3p	-0/16	0/11	0/19	**0/04**	14361
hsa-miR-625-3p	0/04	0/73	0/08	0/57	889
hsa-miR-664a-3p	-0/19	0/06	-0/09	0/44	140
hsa-miR-199b-3p	-0/15	0/11	0/19	0/03	14315

significant p-values are in bold style. Mean Exp., mean read count expression; logFC, log Fold Change.

### Survival analysis

**Fig 3:**
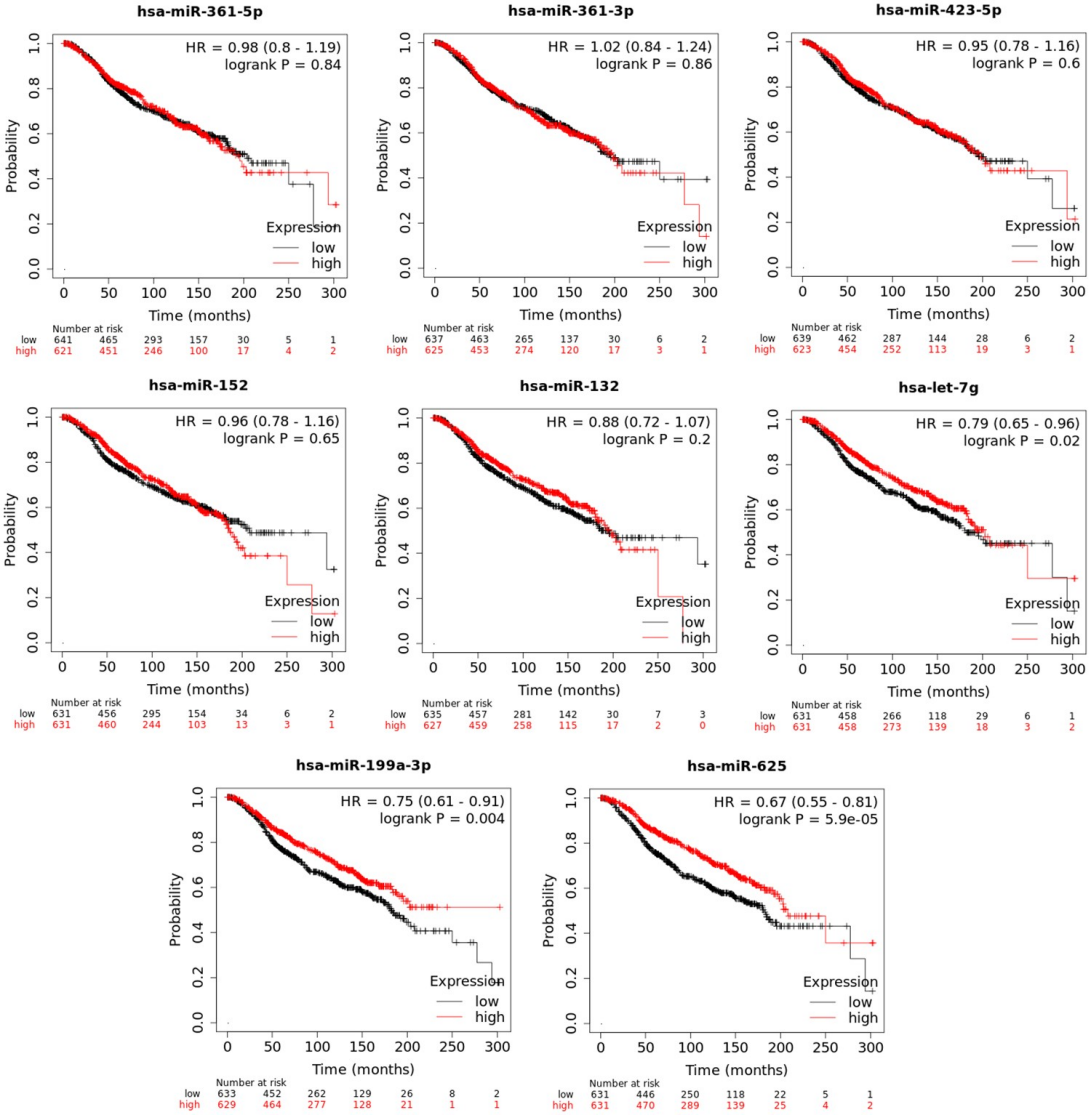
Overall survival Kaplan-Meier plots of top 10 most stable miRNAs. The dataset is METABRIC (n = 1262). Low- and high-risk groups were split based on median expression. Data for hsa-miR-664a-3p and hsa-miR-199b-3p were not available in the METABRIC dataset.

Kaplan-Meier survival analysis was carried out on METABRIC breast cancer dataset (n = 1262) for each of 10 most stable miRNAs. As it is shown in Table 5, three of them had significant association with overall survival: hsa-miR-625-3p (p≤0.00005), hsa-miR-199a-3p (p≤0.004) and hsa-let-7g-5p (p≤0.02). Data for hsa-miR-664a-3p and hsa-miR-199b-3p were not available in the METABRIC dataset. Detailed Kaplan-Meier plots are shown in Fig 3.

**Table 5:** Association with overall survival (OS) on METABRIC dataset.

	**log-rank p**	**HR**
hsa-miR-361-5p	0/84	0/98
hsa-miR-361-3p	0/86	1/02
hsa-miR-423-5p	0/6	0/95
hsa-miR-152	0/65	0/96
hsa-miR-132	0/2	0/88
hsa-let-7g-5p	**0/02**	0/79
hsa-miR-199a-3p	**0/004**	0/75
hsa-miR-625	**0/000059**	0/67

HR, Hazard Ratio.

### miR-361-5p comparison with miR-16-5p on TCGA data

**Fig 4:**
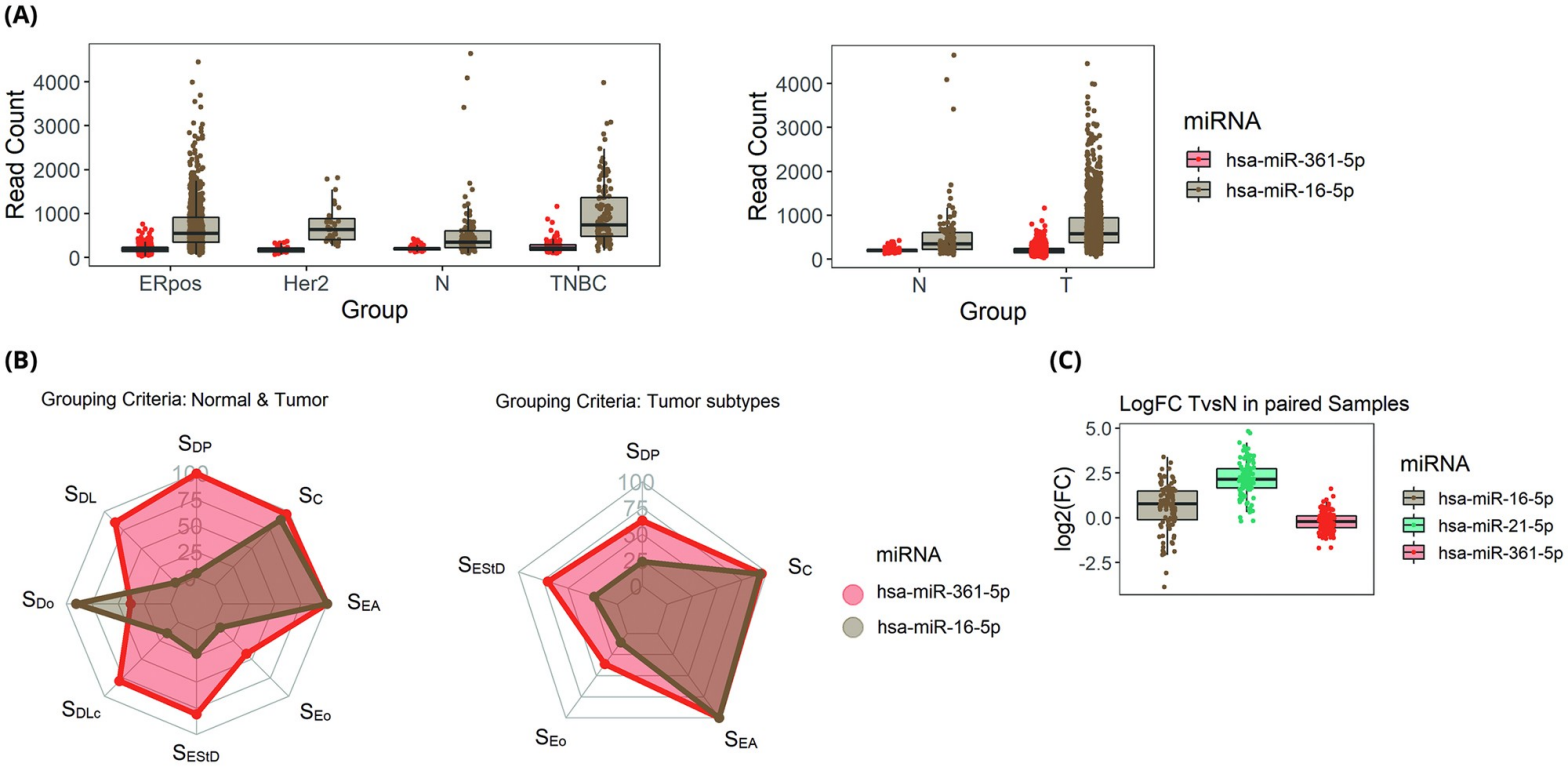
Comparison between hsa-miR-361-5p and hsa-miR-16-5p. (A) Expression level of hsa-miR-361-5p and hsa-miR-16-5p in two different grouping criteria based on TCGA data. T stands for Tumor; N, Adjacent Normal; ERpos, Estrogen Receptor Positive; Her2, Her2 Receptor overexpressed; TNBC, Triple Negative Breast Cancer. (B) Radar plot of scoring components of the multi-component scoring system. To make the plot more compact, following components are averaged. SEo: SEoL, SEoH; SDo: SDoO SDoU; SC: SCr, SCp. (C) Log2 Fold Change of Tumor Vs Normal paired samples based on TCGA data for candidate reference miRNAs along with hsa-miR-21-5p as a well-known oncomiR.

As a promising IC for breast cancer qPCR experiments, hsa-miR-16-5p is a well-known candidate. However, according to our ranking system it was ranked 99th among 185 short-listed miRNAs. Its detailed scores are exhibited in Fig 1. Fig 4A depicts its expression level (in CPM) beside hsa-miR-361-5p, showing hsa-miR-361-5p’s lower standard deviation. Fig 4B illustrates that hsa-miR-361-5p had a higher score in most of our scoring components. As a result of differential expression analysis on tumor vs normal samples, hsa-miR-16-5p was significantly differentiated (adj.p.value: 1.6e-13 lfc: 0.62) while hsa-miR-361-5p showed minor differentiation (adj.p.value: 0.05 lfc: -0.13). As displayed in Fig 4C, hsa-miR-361 had lower abs (LFC) compared to hsa-miR-16-5p in paired tumor and normal samples. Overall, based on our analysis of TCGA miRNA-Seq data, hsa-miR-361-5p is more stable and reliable IC than hsa-miR-16-5p.

### Experimental validation using qRT-PCR

**Fig 5:**
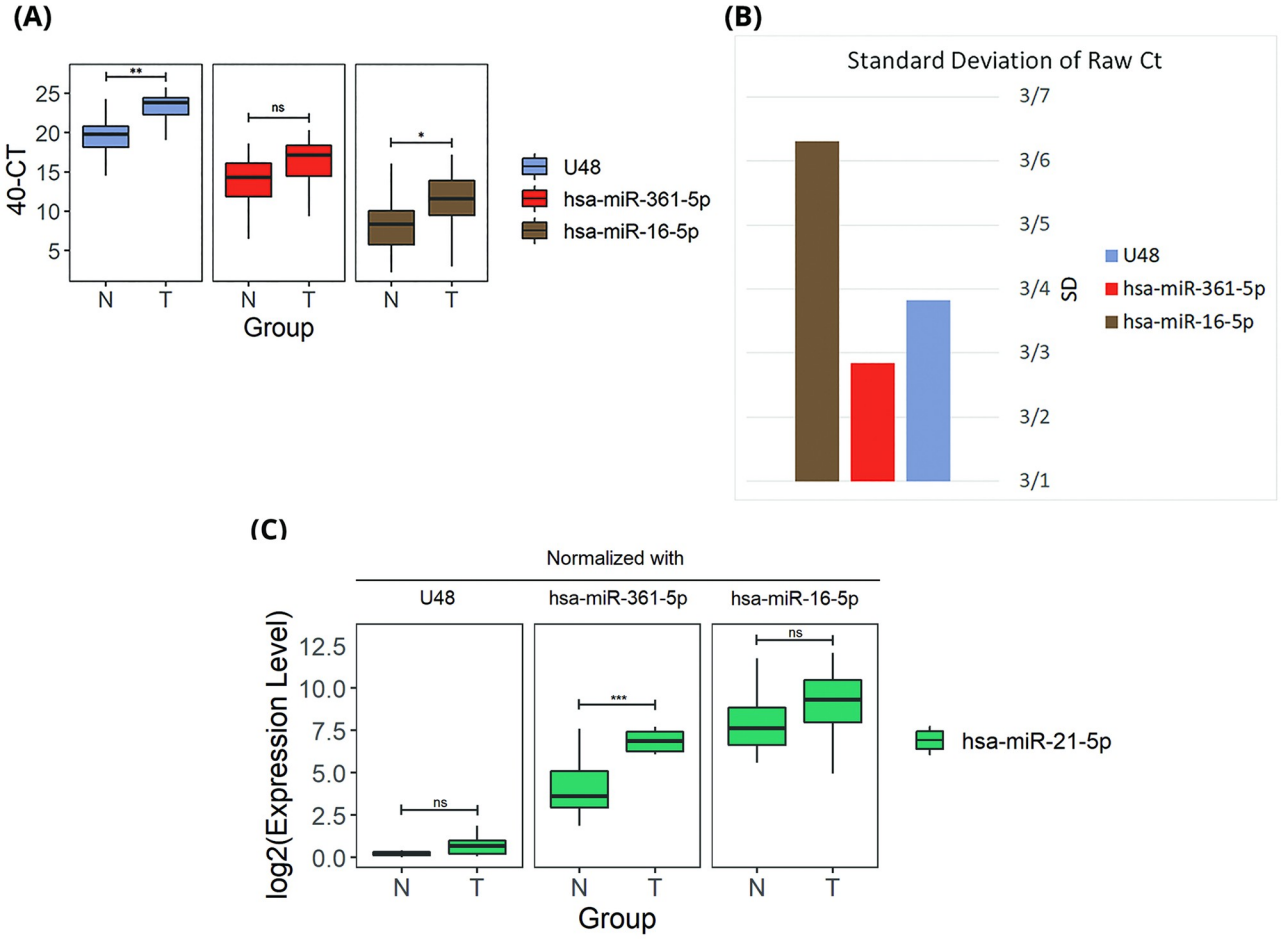
qRT-PCR validation. (A) qRT-PCR raw 40-Ct values of candidate internal controls. The qRT-PCR was ran for 40 cycles so 40-Ct is a measure of expression level. (B) Standard Deviation of candidate internal controls based on raw Ct values of qRT-PCR (C) Expression level of hsa-miR-21-5p normalized with candidate internal controls based on qRT-PCR data. ns: not significant differentiation in Tumor vs Normal samples; ***: p-value<0.001.

A profile of 34 paired samples for breast cancer tumor and adjacent normal tissues were assessed by qRT-PCR to validate hsa-miR-361-5p as a promising IC. We examined the expression of hsa-miR-361-5p, hsa-miR-16-5p, as well as U48 control gene. hsa-miR-16-5p was not detected in two samples, so we excluded them from our analysis. U48 and miR-361-5p had a higher expression level in comparison with miR-16-5p (Fig 5A). Among them, U48 was the most abundant control with a median Ct of 26.64. Standard deviation of raw Ct values was used as a stability measure. Fig 5B shows that hsa-miR-361-5p had the lowest standard deviation.

### Effect on relative expression of hsa-miR-21-5p

hsa-miR-21-5p is one of the most up-regulated miRNAs in breast cancer [37]. In order to evaluate our candidate IC, we measured hsa-miR-21-5p’s expression level on 12 pairs of tissues randomly, using hsa-miR-361-5p, hsa-miR-16-5p as well as U48. As presented in Fig 5C, hsa-miR-361-5p was the only one which could detect hsa-miR-21-5p’s up-regulation, while there was no significant change in hsa-miR-21-5p when normalization was done with U48 or hsa-miR-16-5p.

## Discussion

Since their discovery, miRNAs emerged as important molecules in cancer initiation, progression and pathogenesis [8]. Similar to mRNA expression analysis, qPCR for miRNA quantification requires proper normalization strategies to compensate any non-biological variations [38]. Internal reference genes are currently used as the most universal and accurate method of normalization in qRT-PCR studies [5]. Reference genes might ideally have constant and high expression levels under various circumstances in almost all tissue types. However, such a reference gene does not exist [6]. Luckily, this is not a serious issue since most experimental designs are restricted to few tissue or different histological types of the same tissue.

Different RNA species, including rRNAs, tRNAs, snRNAs, and miRNAs have previously been used as ICs in miRNA real-time q-PCR studies of breast cancer. However, researchers are concerned about their use in normalization, mostly due to their very high expression levels as well as some biases in their stability [14, 39, 40].

Several miRNA expression analysis studies on different tissues have used miRNAs like miR-16 to normalize the expression of interested miRNAs. However, there are controversies about the suitability of these miRNAs as a good normalizer. Mattie et al. used miR-16 and let-7 to normalize miRNA expression on breast tissue sample. Later, Davoren et al. demonstrated their stable expression in malignant, benign, and normal breast tissues [11, 41, 42]. Also, Early studies on miRNAs expressions in breast cancer utilized miR-16 as a normalizer [43, 44]. Interestingly, there are evidence that miR‐15a/16‐1 is involved in the regulation of cell proliferation, apoptosis and invasion [45]. In two other studies, it is reported that miR-16 was significantly downregulated in malignant prostate and breast tissues [17, 46].

To our knowledge there has been only one study that had been miRNA-Seq data to detect promising ICs for breast cancer qRT-PCR studies, in which let-7i-5p and miR-361-5p have been suggested as a suitable one. However, they have used only three criteria to select the best reference miRNAs: mean expression, coefficient of variation and expression fold change between normal & tumor samples [47]. The approach doesn’t take into account other features like clinical or pathologic aspects and also miRNA differentiation between subtypes of tumor samples which are commonly investigated in many cancer studies.

Here, we have discovered the most stable and reliable miRNA ICs for breast cancer qRT-PCR studies, based on TCGA miRNA-Seq data using a multi-component scoring system. This system not only considers the expression stability of miRNAs in different biological groups like tumor subtypes but also checks out for correlation with clinical features of samples.

We investigated TCGA miRNA-Seq breast cancer data for most stable miRNAs based on two sample grouping criteria. First Tumor & Normal samples, second Tumor Subtypes. This approach not only considered tumor-normal differentiation but also within tumor samples variation due to subtypes of breast cancer. This is important because lots of breast cancer experiments are focused on tumor subtypes. After applying our scoring system, top 10 most stable miRNAs were selected for further investigation.

hsa-miR-361-5p had the highest stability score among well-expressed miRNAs, followed by hsa-miR-361-3p and hsa-miR-423-5p. hsa-miR-361-5p ranked first in both grouping criteria showing its convenience for both paired samples and tumor subtype studies.

There are few reports on miR-361-5p role in breast cancer. Zhan, et al., used miRNA-Seq data to detect stably expressed miRNAs in 14 human tumor types. hsa-miR-361-5p was reported as a candidate reference miRNA in eight of 14 cancer types including breast cancer [47]. On the other hand, there are some reports on down-regulation of miR-361-5p in breast cancer [48] and also TNBC subtype [49, 50]. However, both researches have used U6 small nuclear RNA as normalizer for their qRT-PCR validation, which is argued to be up-regulated in breast tumor tissue itself and could potentially lead to data-misinterpretation in breast cancer qRT-PCR experiments [15]. It has also not escaped our notice that, as exhibited in Table 4, hsa-miR-361-5p had some down-regulation in TCGA paired samples, however, its fold change was low (1.2 for normal vs tumor). Moreover, while in our analysis hsa-miR-361-5p had no significant association with overall survival, in one study hsa-miR-361-5p has been reported as a prognostic biomarker for disease free survival, specifically in TNBC subtype [49].

To compare hsa-miR-361-5p usefulness with a commonly used IC, we compared hsa-miR-16-5p expression in the same samples. hsa-miR-361-5p had a better performance in all scoring components except for S_Do_ which is related to the expression of outliers in paired samples. We sat the lower and upper 10% of all samples as outliers.

To validate the suitability of hsa-miR-361-5p as an IC, we performed a qRT-PCR experiment on 34 pairs of tumor and adjacent normal tissue samples. We then quantified hsa-miR-361-5p along with two commonly used ICs, hsa-miR-16-5p and U48. hsa-miR-361-5p had lower standard deviation compared to the others and thus turned out to be a better IC for normal and tumor tissues of breast.

One of the main methods to evaluate the suitability of an IC is to test its performance as a normalizer for quantification the expression of a well-examined gene or miRNA. hsa-miR-21-5p is an oncomiR which is up-regulated in almost all tumor samples [37]. This is further confirmed based on our analysis on TCGA dataset, as shown in Fig 4B. We examined the performance of hsa-miR-361-5p, hsa-miR-16-5p and U48 as an IC and to assess how they can affect the expression level of hsa-miR-21-5p in tumor samples. Surprisingly, hsa-miR-361-5p was the only IC capable of showing the significant up-regulation of hsa-miR-21-5p in our real-time experiment. Moreover, while U48 was a stable reference gene in tumor and normal samples, it could not efficiently detect the significant up-regulation of hsa-miR-21 in tumor samples.

There have been two limitations to our study that should be considered. First, in the qRT-PCR validation phase, most of our tumor samples subtypes were luminal A (64%) and second, the number of samples for evaluating hsa-miR-21-5p expression was low (12 pairs).

In Conclusion, we first introduced the top most stable miRNAs in breast cancer and normal tissues, using a multi-component scoring system on TCGA miRNA-Seq data. This system takes into account the expression stability along with clinical and pathological characteristics of samples. Secondly, we validated that hsa-miR-361-5p is a promising IC for breast cancer qRT-PCR studies and compared it with two commonly used ICs to show its superiority.

## Supporting information

S1 Table Clinical and pathological data on malignant tumor samples where available. T, N and M refer to the primary tumor size, nodal status and distant metastases status according to the TNM breast cancer classification system. ER: = estrogen receptor status; PR: = progesterone receptor status and HER2 = v-erb-b2 erythroblastic leukemia viral oncogene status. Samples marked with star are those checked for miR-21-5p expression.
(DOCX)

S1 Fig General workflow. (TIF)
