# Supplementary material for: miR-361-5p as a promising qRT-PCR internal control for tumor and normal breast tissues
Source: PLoS One. 2021 Jun 8;16(6):e0253009. doi: 10.1371/journal.pone.0253009 (PMC8186776; doi:10.1371/journal.pone.0253009)
Supplement: S1 Table — T, N and M refer to the primary tumor size, nodal status and distant metastases status according to the TNM breast cancer classification system. ER: = estrogen receptor status; PR: = progesterone receptor status and HER2 = v-erb-b2 erythroblastic leukemia viral oncogene status. Samples marked with star are those checked for miR-21-5p expression. (DOCX) [file pone.0253009.s001.docx]

**S1 Table. Clinical and pathological data on malignant tumor samples where available**

| Patient number | Patient age | ER | PR | HER2 | T | N | M | Tumor Grade | Tumor Stage |
| --- | --- | --- | --- | --- | --- | --- | --- | --- | --- |
| 1 | 65 | N | N |  |  |  | 0 |  |  |
| 2 | 47 | N | N | N | 1 | 0 | 0 | 3 | 1 |
| *3 | 50 | P | P | N |  |  | 0 |  |  |
| 4 | 47 | P | P | N | 3 | 0 | 0 | 1 | 2B |
| 5 | 41 | P | P | N | 1 | 0 | 0 | 2 | 1 |
| 6 | 42 | P | P |  | 2 | 2 | 0 | 3 | 3A |
| 7 | 48 | P | P | P |  |  | 0 |  |  |
| 8 | 66 | P | N | N | 2 | 0 | 0 | 2 | 2A |
| *9 | 26 | N | N | N | 2 | 1 | 0 | 3 | 2B |
| *10 | 40 | P | P | N | 1 | 0 | 0 | 2 | 1 |
| 11 | 61 | P | P | N | 2 | 2 | 0 | 2 | 3A |
| *12 | 58 | N | N | P | 1 | 0 | 0 |  | 1 |
| *13 | 67 | P | P | N | 2 | 0 | 0 | 2 | 2A |
| 14 | 55 | P | P | N | 1 | 0 | 0 | 2 | 1 |
| *15 | 52 | P | P |  | 2 | 2 | 0 | 3 | 2A |
| *16 | 37 | P | P | N | 2 | 0 | 0 | 2 | 2A |
| 17 | 44 | P | P |  | 3 | 1 | 0 | 2 | 3A |
| *18 | 60 | P | P | N | 1 | 0 | 0 | 2 | 1 |
| *19 | 49 | P | P | N | 1 | 2 | 0 | 1 | 3A |
| 20 | 46 | P | P | N | 2 | 0 | 0 | 3 | 2A |
| 21 | 60 | N | N | P | 2 | 0 | 0 | 2 | 2A |
| 22 | 39 | P | P | N | 3 | 2 | 0 | 3 | 3A |
| 23 | 50 | P | P | N | 2 | 1 | 0 | 2 | 2B |
| *24 | 63 | N | N | N | 1 | 0 | 0 | 3 | 1 |
| 25 | 50 | P | P | N | 1 | 3 | 0 | 2 | 3C |
| *26 | 65 | P | P | N | 1 | 0 | 0 | 1 | 1 |
| *27 | 35 | P | P | N |  |  | 0 | 3 |  |
| 28 | 72 | P | P | N | 2 | 0 | 0 | 2 | 2A |
| 29 | 50 | P | P | N |  |  | 0 | 3 |  |
| 30 | 35 | P | P | P | 2 | 0 | 0 | 2 | 2A |
| 31 | 50 | P | P | N | 1 | 1 | 0 | 2 | 2A |
| 32 | 45 | P | P | N |  |  | 0 |  |  |
| 33 | 46 | P | P | N | 1 | 1 | 0 | 1 | 2A |
| 34 | 55 | P | P | N | 1 | 0 | 0 | 2 | 1 |

T, N and M refer to the primary tumor size, nodal status and distant metastases status according to the TNM breast cancer classification system. ER: = estrogen receptor status; PR: = progesterone receptor status and HER2 = v-erb-b2 erythroblastic leukemia viral oncogene status. Samples marked with star are those checked for miR-21-5p expression.
