# Supplementary figures and images for: miR-361-5p as a promising qRT-PCR internal control for tumor and normal breast tissues
Source: PLoS One. 2021 Jun 8;16(6):e0253009. doi: 10.1371/journal.pone.0253009 (PMC8186776; doi:10.1371/journal.pone.0253009)

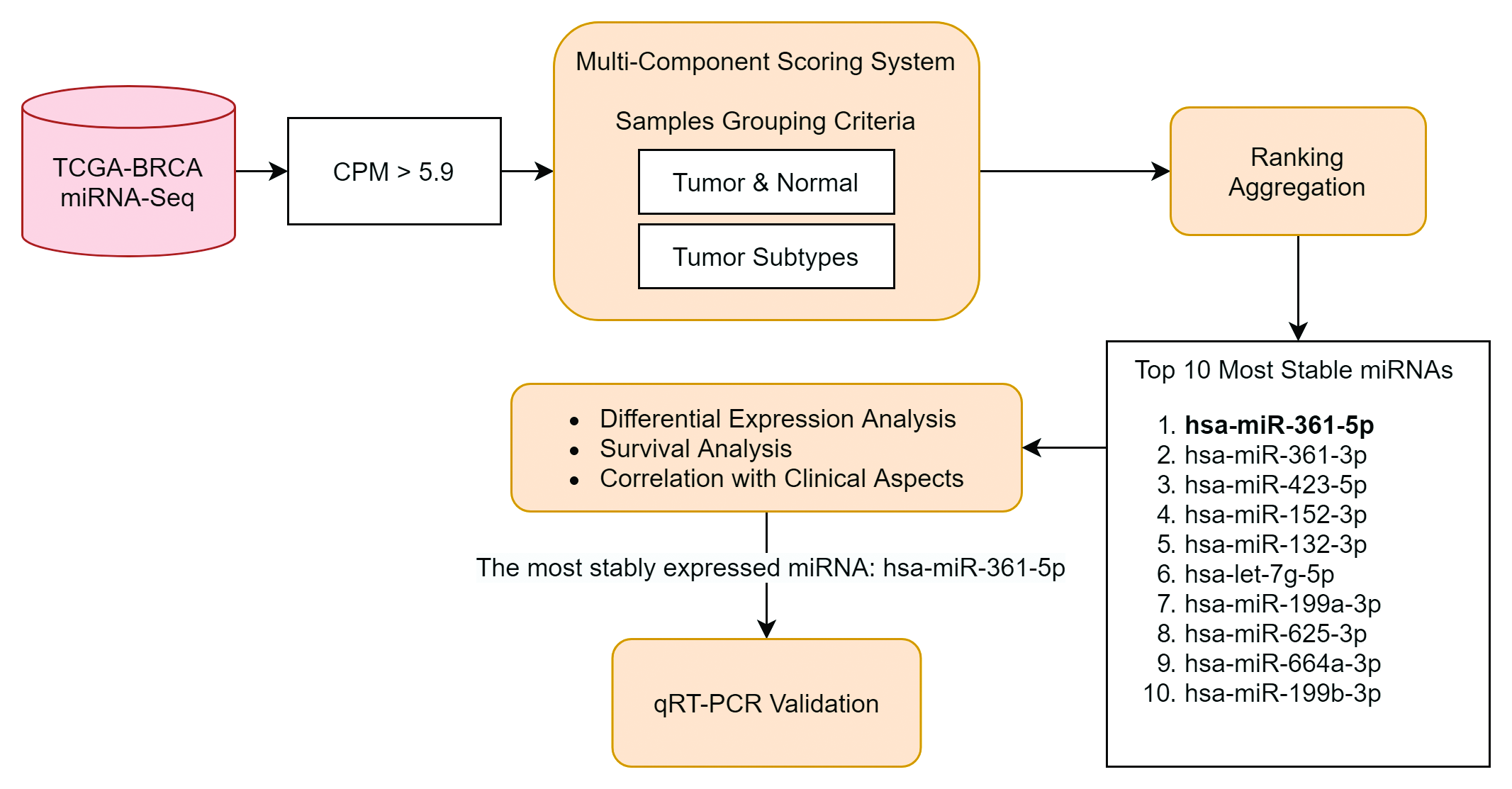

Supplement: S1 Fig — (TIF) [file pone.0253009.s002.tif]
